# Gut Metabolite Trimethylamine-N-Oxide in Atherosclerosis: From Mechanism to Therapy

**DOI:** 10.3389/fcvm.2021.723886

**Published:** 2021-11-23

**Authors:** BingYu Wang, Jun Qiu, JiangFang Lian, Xi Yang, JianQing Zhou

**Affiliations:** ^1^Department of Cardiology Vascular Internal Medicine, Ningbo Medical Center LiHuiLi Hospital, Ningbo University, Ningbo, China; ^2^Central Laboratory, Ningbo Institute of Innovation for Combined Medicine and Engineering, Ningbo, China

**Keywords:** TMAO, L-carnitine, atherosclerosis, endoplasmic reticulum stress, vascular calcification

## Abstract

Atherosclerosis is associated with various pathological manifestations, such as ischemic heart disease, ischemic stroke, and peripheral arterial disease, and remains a leading cause of public health concern. Atherosclerosis is an inflammatory disease characterized by endothelial dysfunction; vascular inflammation; and the deposition of lipids, cholesterol, calcium, and cellular debris within the vessel wall intima. In-depth studies of gut flora in recent years have shown that bacterial translocation and the existence of bacterial active products in blood circulation can affect the inflammatory state of the whole blood vessel. The gut flora is considered to be a large “secretory organ,” which produces trimethylamine-N-oxide (TMAO), short-chain fatty acids and secondary bile acids by breaking down the ingested food. Studies have shown that TMAO is an independent risk factor for the occurrence of malignant adverse cardiovascular events, but whether it is harmful or beneficial to patients with cardiovascular diseases with mild or no clinical manifestations remains controversial. We review the relationship between TMAO and its precursor (L-carnitine) and coronary atherosclerosis and summarize the potential molecular mechanism and therapeutic measures of TMAO on coronary atherosclerosis.

## Introduction

Atherosclerosis (AS) is a disease of large- and medium-sized arteries and is characterized by endothelial dysfunction, vascular inflammation, and the buildup of lipids, cholesterol, calcium, and cellular debris within the vessel wall intima. It is a chronic inflammatory condition that involves complex interactions among lipids, vascular endothelial cells, and immune cells as well as smooth muscle cells. Although there are many effective ways to treat cardiovascular diseases, it is still one of the leading causes of death worldwide (1). Extensive research on intestinal flora in recent years has shown that bacterial translocation and the existence of bacterial active products in the blood circulation can aggravate the inflammatory state of the whole blood vessel. These changes in the composition of the intestinal microbiota have been associated with diseases such as atherosclerosis, hypertension, heart failure, chronic kidney disease, obesity, and type 2 diabetes (2). The gut microbiome functions as an endocrine organ and generates bioactive metabolites that can impact host physiology.

The classic product trimethylamine oxide (TMAO) is a phospholipid metabolite related to intestinal microorganisms and is closely related to people's everyday diet (3). Circulating TMAO arises from endogenous liver and gut production and exogenous food intake. Generally, plasma TMAO has been measured measured using the ultra-performance liquid chromatography-tandem mass spectroscopy method (4) and a simple isocratic high-throughput liquid-chromatography tandem mass-spectrometry method (5). Nuclear magnetic resonance spectroscopy has also been used to quantify urinary trimethylamine (TMA) and TMAO (6).

Hazen et al. first discovered plasma intestinal flora-dependent small molecule metabolic spectrum by metabonomics and found that the plasma levels of TMAO and its precursors, choline and betaine, can predict the risk of human cardiovascular disease, thus identifying a novel relationship between intestinal flora and metabolic health (7). TMAO is predominantly sourced from choline (found in foods such as red meat, fish, poultry, and eggs) and metabolized by microbiota to TMA and then to TMAO through the action of the hepatic enzyme, flavin monooxygenase 3 (FMO3) (8). However, recent literature has shown that TMAO not only passes through the FMO3 oxidation system of the liver but also through an oxidation pathway in the intestinal tract (9). Recent studies have reported controversial results regarding the relationship between atherosclerosis and TMAO and its precursor (L-carnitine).

In this paper, we describe in detail how TMAO promotes the transformation of macrophages to foam cells, which lead to reverse cholesterol transport that inhibits bile acid and sterol metabolism, and at the same time enhances the hyperactivity of platelets, which leads to the formation of atherosclerotic plaques. In addition, Chen et al. demonstrated for the first time that TMAO can bind and activate PERK to cause atherosclerosis, which provides a new mechanism of action of TMAO-induced atherosclerosis, which will be described in detail below (10).

## Effect of TMAO and Its Precursor (L-CARNITINE) on Atherosclerosis

### TMAO and Atherosclerosis

Atherosclerosis is the pathological basis of coronary atherosclerotic heart disease (11). Clinically, coronary atherosclerotic heart disease (CHD) is divided into chronic coronary artery disease (CAD) and acute coronary syndrome [i.e., unstable angina, non-ST-segment elevation myocardial infarction (NSTEMI), ST-segment elevation myocardial infarction (STEMI), and sudden coronary death] (12). Recent studies in animal models and human subjects have demonstrated a close relationship between the gut microbiota and atherosclerosis, especially the gut metabolite TMAO (13–15).

#### TMAO and Chronic Coronary Artery Disease

In recent years, several studies have suggested that TMAO is a new independent risk factor for atherosclerosis (Table 1). Plasma levels of TMAO are reportedly positively associated with cardiovascular risk and mortality in a dose-dependent manner (16).

**Table 1:** The negative effects of TMAO on atherosclerosis demonstrated *via* clinical and non-clinical experiments.

**Effect**	**Study type**	**Type of study**	**Study population**	**Feed/ treatment reagent**	**Follow-up time**	**Number of clinical studies**	**Conclusion**	**Limitation**	**References**
Negative effects	Human	Prospective cohort study	671	—	1 year	2	There is a hierarchical relationship between TMAO levels and the risk of subsequent cardiovascular adverse events in patients with prior ischemic stroke.	The follow-up time for this study was 1 year and there was only data from a single research center.	(14)
				—					
		Systematic review	1,622	—	4.95 years on average	6	There was a positive correlation between elevated circulating TMAO levels and a higher risk of death in adults.	The data were incomplete, in the manuscripts, adjustment for these confounders was not available.	(15)
		Meta-analysis	31,230	—		20			
		Systematic review	15,662; 13,944	—	4.3 ± 1.5 years	17;14	There was a significant dose-dependent positive correlation between plasma TMAO levels, cardiovascular events, and mortality.	The distribution of TMAO levels in the general population is currently unknown and “standardized normal values” are not currently available.	(16)
		Meta-analysis	—			__			
		Historical independent controlled cohort study	100	—	2 years	2	Plasma TMAO was significantly associated with new atherosclerosis and plaque rupture in VLST patients.	The study was lack of a control group consisting of patients with a similar duration of stent implantation without VLST and the data is from a signal center.	(32)
		Case-control study	2,595	—	24 h (carnitine attack test)	__	In patients with high levels of plasma TMAO, excessive intake of L-carnitine can predict CVD and MACE.	It lacks research on low concentrations of TMAO on human diseases.	(35)
	Animal	C57B/L6 and NZW / LacJ mice	—	1.06% choline	__	__	The area of atherosclerotic plaque in mice on a high choline diet was larger.	The study don't use germ-free mouse genotypes suitable to explore the influence of the gut flora.	(14)
		ApoE ^−/−^ and Ldlr ^−/−^ mice	—	3%choline	__	__	There was no direct correlation between plasma TMAO and the degree of atherosclerosis.	This study lacks experimental data on humans.	(18)
		Plaque instability mice model					Plasma levels of TMAO are associated with atherosclerotic plaque instability.		
		Female, C57BL/6J and Apoe ^−/−^ mice	—	1.3% L-carnitine	__	__	The metabolism of the intestinal flora of L-carnitine (a trimethylamine in which red meat is rich) can also produce TMAO and accelerate atherosclerosis in mice.	The mouse genotype used is single.	(38)

*VLST, very late stent thrombosis; MACE, Major adverse cardiac events; CVD, Chronic cardiovascular disease; HD, Hemodialysis disease*.

The higher the concentration of TMAO in plasma, the greater the probability of malignant cardiovascular and cerebrovascular events (13–15). A recent prospective case-control study (17) showed that in a 10-year period, regardless of the level of baseline TMAO, the final increase in TMAO was significantly associated with an increased risk of CHD, and the TMAO–CHD relationship could be improved by dietary changes (17). However, there are many confounding factors in the process of a 10-year investigation. Although substantial evidence suggests that an increase in the level of TMAO is associated with the risk of cardiovascular diseases including atherosclerosis, chronic kidney disease, and hypertension, the direct effect of TMAO on vascular endothelial function remains unclear. However, some studies have reported different points of view (Table 2). Koay et al. compared healthy (high-fiber) and unhealthy (high-cholinergic) diets and found that the TMAO produced by the diet is only partially related to the degree of atherosclerosis, because a high-cholinergic diet may work through a series of pro-inflammatory and platelet activations, rather than single mediators (18). Besides, Jaworska et al. suggested that TMAO is not a toxic molecule of cardiovascular disease, rather its metabolic precursor TMA is (19, 20). Under a normal diet, C57BL/6J and CD-1 mice with elevated TMAO levels showed no increase in cardiac injury indicators (21). Low-dose TMAO treatment can reduce plasma N-terminal pro-B-type natriuretic peptide and vasopressin, left ventricular end-diastolic pressure, and cardiac fibrosis in pressure-overloaded hearts in hypertensive rats (22). The levels of plasma lipids and lipoproteins do not change with the level of TMAO, and TMAO can delay the formation of aortic lesions in *ApoE*^−/−^ mice (23). However, this speculation lacks multi-center and multi-dimensional clinical research. Plasma TMAO levels are affected by many factors including species specificity, growth environment, diet, and intestinal microbes. Hence, further studies investigating the effects of different species and different diets on the contents of TMAO, the intracellular concentration of TMAO, and other proteins are needed to determine the role of TMAO in human health and diseases.

**Table 2:** The positive or non-effects of trimethylamine-N-oxide (TMAO) on atherosclerosis demonstrated *via* clinical and non-clinical experiments.

**Effect**	**Study type**	**Type of study/mouse model/cell model**	**Study population**	**Feed/treatment reagent**	**Follow-up time**	**Conclusion**	**Limitation**	**References**
Positive or non-effect	Human	Case-control study	20	–	24 weeks	Oral administration of l-carnitine significantly increased plasma TMAO, but no changes in lipid profile or other markers of adverse cardiovascular events were detected within 24 weeks.	The study only detected changes in cardiac markers within 24 weeks after L-carnitine intervention.	(43)
		Nested case-control study	1,879	–	10 years	TMAO and L-carnitine levels were not associated with the occurrence of atrial fibrillation and heart failure.	The study has a single population and a single method of measuring metabolites.	(44)
		Case self-control study	31	–	6 months	Oral administration of l-carnitine causes elevated TMAO levels, but may be beneficial for vascular damage in HD patients.	The research object is HD patients, and there is interference from other urinary toxins.	(45)
	Cell	Rat cardiomyocytes (H9c2 cell line)	–	Different concentrations of TMA and TMAO treatment	__	TMA instead of TMAO may be a sign of toxins and cardiovascular disease.	The study is only for patients with aortic stenosis.	(19)
	Animal	Male, Sprague-Dawley and Wistar-Kyoto rats.	–	Plasma levels of TMA and TMAO were measured	__	TMA rather than TMAO may be a marker and modulator of cardiovascular risk.	Due to the effect of age on plasma TMAO, the rats were only 4 months and 10 months. It need older rats.	(20)
		C57BL / 6J,CD-1 Fmo1^−/−^, 2^−/−^, and 4^−/−^ mice	–	a standard chow diet	__	Under normal dietary conditions, TMAO does not increase plasma cholesterol or act as a pro-atherosclerotic molecule.	There is little research on the substances that produce TMAO precursors.	(21)
		Male, spontaneously Hypertensive rats (SHR) and Normotensive Wistar142 Kyoto rats (WKY)	–	TMAO treatment at different concentrations (1 g/L, 0.333 g/L, 0.1 g/L)	__	A moderate increase in plasma TMAO did not negatively affect the circulatory system. In contrast, increasing TMAO in the diet seems to reduce the diastolic dysfunction of the stress-overloaded heart.	The study was performed on one animal model of hypertension and HF.	(22)
		Male ApoE^−/−^ mice (HCETP)	–	L-carnitine and/or mexanazole (a flavin monooxygenase 3 inhibitor)	__	TMAO slowed down the formation of aortic lesions in this mouse model, and was found to have a potential protective effect on the development of human atherosclerosis.	The animal model used in this study is a special gene knockout model, which is not well-representative.	(23)

*HD, Hemodialysis disease*.

#### TMAO and Acute Coronary Syndrome (ACS)

More than half of cardiovascular deaths are caused by ACS, and the main cardiovascular disorder causing these acute cardiovascular events is the development of atherosclerosis (24). TMAO is reported to be involved in vascular calcification and plaque instability, which are the key risk factors for malignant cardiovascular events (25). In 2017, Li et al. proposed that TMAO levels among patients presenting with chest pain can predict both short- and long-term risks of incident cardiovascular events (26). Two prospective cohorts (27) found that plasma TMAO levels are associated with a high coronary atherosclerotic burden in patients with STEMI (27). Tan et al. (28) and Gao et al. (29) also reached a similar conclusion. Subsequently, Matsuzawa et al. found that high TMAO levels are associated with chronic coronary plaque progression. Chronic, but not acute, phase TMAO level is a significant and independent predictor of future cardiovascular events after STEMI (30). In addition, plaque rupture and secondary formation of thrombi play important roles in ACS development (31). Multivariate analysis showed that plasma TMAO was significantly associated with new atherosclerosis and plaque rupture in patients with very late stent thrombosis (VLST) (32). However, TMAO was not significantly correlated with 30-day left ventricular systolic dysfunction in patients with a first anterior STEMI after primary revascularization (33).

In summary, TMAO is an independent risk factor for the risk estimation of malignant cardiovascular disease. However, whether it is harmful or beneficial for subjects with mild cardiovascular diseases or even those without clinical manifestations is still controversial, likely because of the following reasons: (i) similar strains of mice in different studies from different suppliers differ in baseline bacterial gut microbiome, diet (e.g., different choline levels), age of the mice, animal housing conditions, and duration of the experiment. (ii) In human studies, this includes differences in race and patient age, as well as sex, diet, and drug use, which can cause changes in the composition of the gut bacterial microbiota and alter the relationship between plasma TMAO levels and atherosclerosis. (iii) Most importantly, TMAO may be just one of many factors that influence atherosclerosis and its complications. Further, depending on the impact size of each factor, TMAO may be more or less dominant.

### L-Carnitine (the Precursor of TMAO) and AS

**Figure 1:**
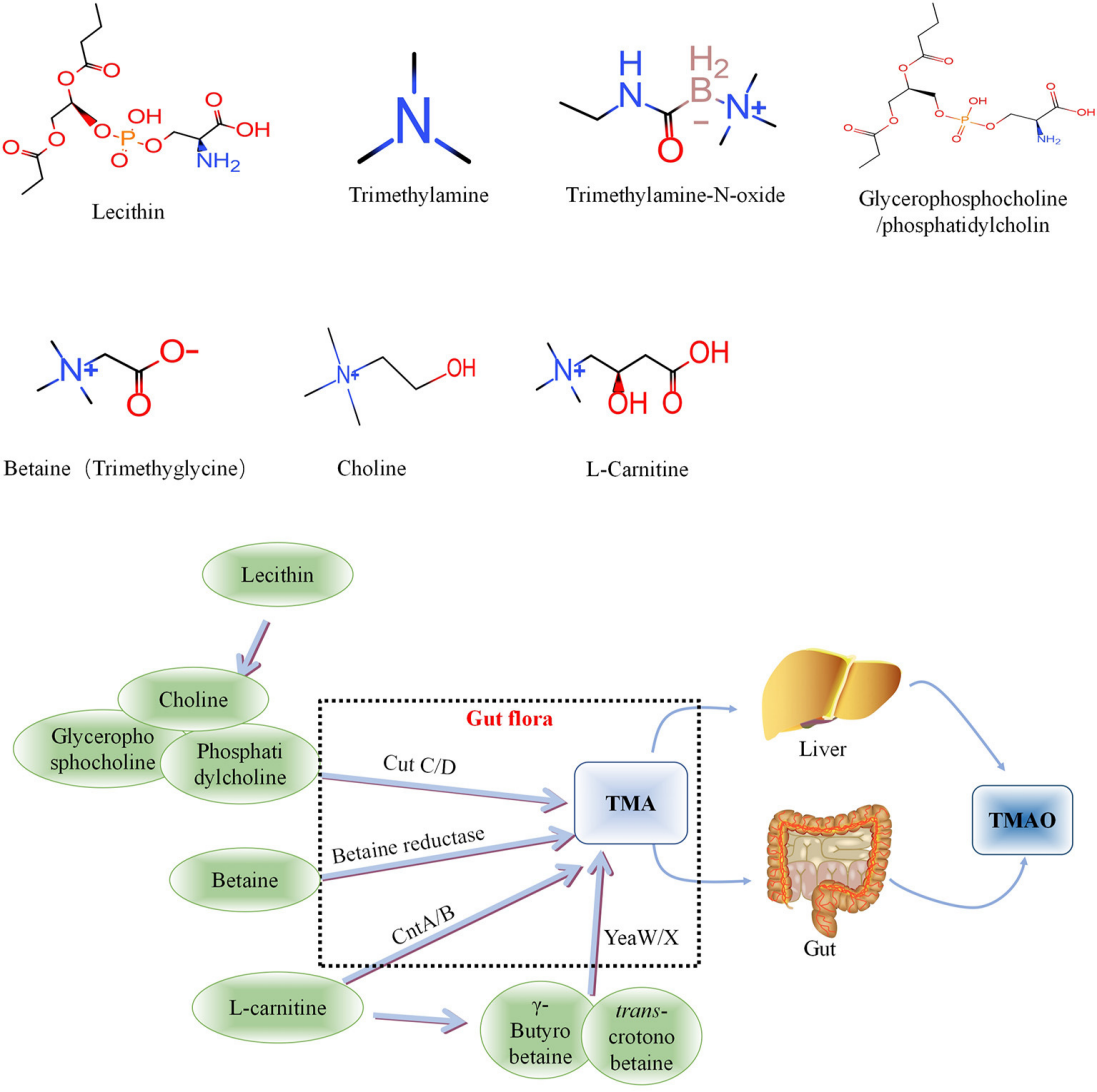
Metabolic processes of Trimethylamine-N-oxide (TMAO) and its precursors *in vivo*. Choline is mainly cleaved by choline trimethylamine-lyase system (CutC/D) of intestinal flora to produce TMA, phosphatidylcholine and glycerophosphorylcholine with similar effects. Betaine can be catalyzed by L-carnitine dehydrogenase and then reduced to TMA by betaine reductase. L-carnitine can be directly transformed into TMA *via* intestinal flora-derived CntA/CntB, and also into γ-butyrobetaine and betaine, and further converted to TMA through the yeaW/yeaX enzyme system of gut flora. TMA can be oxidized to TMAO through the liver and intestines (jejunum and cecum) and enter the blood circulation. TMA, Trimethylamine; TMAO, Trimethylamine-N-oxide.

Intestinal microbial-derived TMAO precursors include choline, phosphatidylcholine, L-carnitine, γ-butyrobetaine, trans-crotonobetaine, and glycerophosphate choline, all of which are abundant in the human diet (34). The metabolic pathways of these precursors are shown in Figure 1.

L-carnitine, a TMA found abundantly in red meat, has aroused considerable research interest because an increasing number of studies have found that it is associated with atherosclerosis (35). L-carnitine can be directly transformed into TMA *via* intestinal flora-derived *CntA/CntB*, and also into γ-butyrobetaine and betaine, and further converted to TMA through the *yeaW/yeaX* enzyme system of intestinal flora (36). The metabolic pathways of the above precursors and the relationship between L-carnitine and TMAO are shown in Figure 1. TMA can be oxidized to TMAO through the liver and intestines (jejunum and cecum) and enter the blood circulation.

In 2013, Koeth et al. evaluated the plasma L-carnitine levels of subjects with high levels of TMAO (*n* = 2,595) and found that the intake of large amounts of L-carnitine caused cardiovascular disease (CVD) and major adverse cardiac events (MI, stroke, or death). In mice with intact gut microbiota, dietary supplementation of TMAO, carnitine, or high choline can significantly reduce the reverse transport of cholesterol in the body, thereby aggravating the progression of atherosclerosis in mice (35). Subsequently, a clinical trial by Koeth et al. (NCT01731236) showed that L-carnitine produced more TMAO in the omnivorous group compared with the vegetarian group, thereby inducing atherosclerosis formation (37). Another multicenter cohort study showed that higher carnitine levels were significantly associated with the presence of carotid plaque and the progression of a larger baseline and mean carotid artery intima-media thickness (38). Kuka et al. transferred the L-carnitine-dependent microbiota of Wistar rats and found that this inhibits the process of intestinal flora-dependent TMAO-induced atherosclerosis (39). In addition, platelet mitochondrial DNA methylation is an emerging innovative biomarker, and lipid profiles and TMAO levels have been measured. A group of elderly women who performed regular physical exercises and received supplemental L-carnitine for 6 months had increased D-cyclomethylation of platelets. The mtDNA methylation involved may be a potential mechanism for exposure to L-carnitine, TMAO, and atherosclerotic biomarkers (40). However, L-carnitine was considered to be a cardioprotective factor in the past. For instance, from the perspective of clinical trial research, Samulak et al. (41), Adeva-Andany et al. (42), and Papandreou et al. (43) showed that L-carnitine has no significant effect on the occurrence of cardiovascular events or the formation of cardiovascular markers. From the perspective of animal experiments, in ApoE^−/−^ mice expressing human cholesteryl ester transfer protein (hCETP), TMAO derived from L-carnitine levels inversely correlated with aortic lesion size in both the aortic root and thoracic aorta (23). Therefore, the specific effect of L-carnitine on the human body is not clear, and it may be related to the amount or method of intake, the time interval between doses, the patient's physique, sex, or ethnicity. Hence, it is speculated that different results of TMAO on CVD may be related to different metabolic precursors (choline, L-carnitine, and betaine). For the complex relationship between TMAO and its metabolic precursors and chronic cardiovascular disease, more rigorous experimental protocols need should be designed to eliminate the interference of different precursor metabolites. Therefore, careful study of different precursor substances can provide more scientific and reasonable guidance for the dietary planning of patients with chronic cardiovascular disease and ACS. However, it is not clear how TMAO functions in patients with coronary atherosclerosis. The following is a summary of the current research progress on the mechanism of TMAO in coronary atherosclerosis, to provide some theoretical basis for subsequent treatment and research.

## The Molecular Mechanism of TMAO Aggravating Coronary Atherosclerosis

### Vascular Dysfunction: TMAO Promotes Oxidative Stress and Inflammation in Endothelial Cells

**Figure 2:**
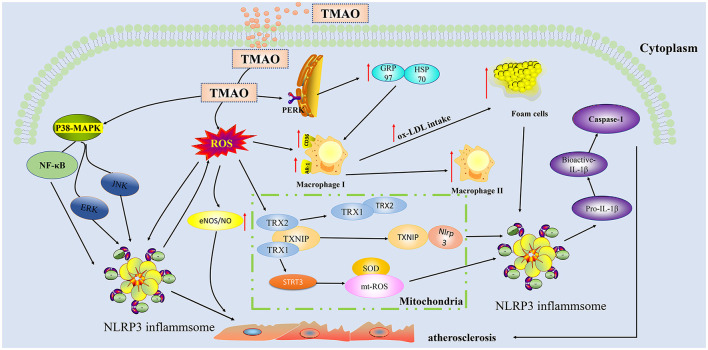
Trimethylamine-N-oxide (TMAO) activates inflammatory and oxidative stress pathways in endothelial cells and aggravates the formation of plaques. TMAO can activate ROS oxygen radicals through indirect pathways and activate TXNIP-NLRP3 and STRI3-mtROS in mitochondria, thereby triggering the production of inflammatory cytokines IL-1β, IL-18, and caspase-1.TMAO can also directly activate p38-MAPK and NF-κB cascading signaling pathways to increase the secretion of NLRP3 in inflammasome, thereby aggravating the injury of endothelial cells. TMAO can act on macrophages to enable ox-LDL to absorb more lipids. TMAO can bind to the PERK receptor of endoplasmic reticulum stress, upregulate GRP97/HSP70, and synergistically increase lipid uptake by macrophages. TMAO, Trimethylamine-N-oxide; ROS, Reactive oxygen species; MAPK, Mitogen-activated protein kinases; PERK, RNA-dependent protein kinase-like endoplasmic reticulum kinase; NLRP3, NOD-like receptor protein 3; ox-LDL, Oxidized low density lipoprotein; SOD2, superoxide dismutase2; GRP97, Glucose-regulated protein 97; HSP70, Heat shock protein 70; eIF2α, eukaryotic Initiation Factor 2 alpha; TRX, Thioredoxin; TXNIP, Thioredoxin-interactive protein; SIRT3, Sirtuin 3; CD36, Cluster Differentiation 36; SR-A1:Scavenger receptor class A type 1.

Vascular dysfunction is an important risk factor for atherosclerotic heart disease (44). Endothelial cells play a central role in maintaining the homeostasis of blood vessel function (45). The pathophysiology of endothelial dysfunction is complex, in that it is based on endothelial nitric oxide synthase uncoupling and endothelial activation following stimulation by various inflammatory mediators (molecular patterns, oxidized lipoproteins, and cytokines) (46). In recent years, extensive research (Figure 2) has shown that TMAO can activate the inflammasome NOD-like receptor protein 3 (Nlrp3) through different pathways to activate the inflammatory signal pathway, resulting in aggravation of oxidative stress and, in turn, of endothelial dysfunction. Apart from Nlrp3 inflammasome, mitogen-activated protein kinases (MAPK) and nuclear factor kappa-light-chain-enhancer of activated B cells (NF-κB) are involved in the pathogenesis of chronic inflammation (47). In particular, the Nlrp3 inflammasome is a cytosolic multiprotein complex expressed in immune/inflammatory cells as well as cardiovascular system cells, including endothelial cells, myofibroblasts/fibroblasts, and cardiomyocytes, which, through the processing and release of interleukin (IL)-1β and IL-18 and the induction of cell death processes (pyroptosis, apoptosis, and necroptosis), plays a key role in the maintenance of host homeostasis and in sustaining the pathophysiological events underlying CVDs (48). Conversely, TMAO can release inflammatory factors (caspase-1, IL-1 β) (49) in a dose-and time-dependent manner by activating the reactive oxygen species (ROS)–thioredoxin-interacting protein (TXNIP) –NLRP3 signaling pathway (50) and inhibiting sirtun3 (SIRT3) –superoxide dismutase2 (SOD2)–mitochondrial ROS signaling pathway (51), thereby causing endothelial cell injury and aggravating the formation and development of atherosclerosis.

MAPKs are a group of conserved serine/threonine protein kinases, including ERK, JNK, and p38. MAPK signaling pathways can participate in the formation of atherosclerosis by regulating the proliferation and migration of vascular endothelial cells (52). TMAO has also been shown to induce inflammatory markers in mice and in human aortic endothelial cells and vascular smooth muscle cells owing to activation of MAPK, extracellular signal kinase, and NF-κB signaling cascade, along with promoting the recruitment of activated leukocytes to endothelial cells.

TMAO affects not only classical inflammatory signaling pathways and inflammasomes but also immune-related cytokines (53, 54). Chen et al. have confirmed that the increase of circulating TMAO downregulates IL-10 and promotes vascular inflammation, contributing to endothelial dysfunction by mouse experiments (55). The above experiments *in vivo* and *in vitro* have confirmed that TMAO can cause inflammation of endothelial cells, and similar results can be observed in humans. In summary, the accumulation of TMAO will not only increase oxidative stress and endothelial cell damage and permeability but also lead to an increase endothelial-derived NOS expression (56), thereby aggravating disease progression.

### Vascular Calcification: TMAO Activates the NLRP3 Inflammasome and NF-κB Signaling Pathway in Vascular Smooth Muscle Cells (VSMCs)

Vascular calcification (VC) is a phenomenon of disseminated deposition of mineral content within the medial layer of arteries. It is now considered to be an active osteogenic process of vascular cells (mainly VSMCs), similar to osteoblast formation, and is defined by the stages of osteogenic differentiation, matrix maturation, and matrix mineralization (57). Zhang et al. found that TMAO promoted calcium/phosphate-induced calcification in the vascular smooth muscle cells of rats and human urinary system patients in a dose-dependent manner, and upregulated the expression of bone-related molecules (Runt-related transcription factor2, Runx2, and Bone morphogenetic protein-2, BMP2). It is suggested that TMAO promotes the osteogenic differentiation of vascular smooth muscle cells. In this process, TMAO activated the NLRP3 inflammasome and NF-κβ signaling pathway (58). Therefore, TMAO may activate these elements to promote vascular calcification. Similarly, another *in vitro* study found that TMAO can also upregulate the NF-κB signaling pathway to promote adipogenic differentiation and inhibit the osteogenic differentiation of bone marrow-derived mesenchymal stem cells (BMSCs) (59).

Another study found that in the aorta of fish protein-fed mice, the atherosclerotic plaque area and degree of calcification were significantly higher than that of casein- or-soy protein-fed mice, but there were no significant changes in lipid-related content. It is worth noting that the concentration of TMAO is ~6 times higher than that of the control group after being fed fish protein; therefore, it is speculated that the degree of arterial calcification may be related to the high concentration of TMAO (60). Li et al. enrolled 179 patients with STEMI to measure the plasma levels of TMAO by using stable isotope dilution liquid chromatography tandem mass spectrometry, and used optical coherence tomography (OCT) to analyze the calcified lesions of 127 patients. The level of TMAO was significantly positively correlated with the incidence of calcification in the intimal lesion (25). Carnitine is the precursor of TMAO; interestingly, Okui et al. performed an unbiased quantitative proteomics and pathway network analysis that identified increased carnitine-O-octanoyltransferase (CROT) levels in calcifying primary human coronary artery smooth muscle cells (SMCs). CROT was identified as a novel contributing factor in vascular calcification *via* promoting fatty acid metabolism and mitochondrial dysfunction (61). Moreover, this study also provides new potential target genes for the clinical treatment of vascular calcification.

Taken together, previous studies show that TMAO can aggravate the process of atherosclerotic plaques by promoting vascular calcification. Nonetheless, whether the specific mechanism of this is related to the transdifferentiation of VSMCs to a bone-like phenotype, loss in SMC lineage markers, enhanced pro-calcific microRNAs, increased intracellular calcium level, apoptosis, or aberrant DNA damage response (DDR) requires further investigation.

### Macrophage Foaming: TMAO Promotes the Conversion of Macrophages Into Foam Cells

As the major immune cells in atherosclerotic lesions, macrophages play a critical role in the development of atherosclerosis. A central hallmark of atherosclerosis is foam cell formation characterized by uncontrolled lipoprotein accumulation within macrophages (62). TMAO has a similar effect on the development of atherosclerosis (Figure 2). TMAO can upregulate scavenger receptors, such as cluster of differentiation 36 (CD36) and scavenger receptor class A type 1 (SR-A1), and promote the formation of foam cells, leading to development and progression of atherosclerosis (7). As mentioned previously, the TMAO is similar to that of oxidized low density lipoprotein (ox-LDL), which can promote the differentiation of monocytes into macrophages that scavenge ox-LDL and transform into foam cells (63). TMAO can also promote endoplasmic reticulum stress (ERS) by up-regulating SR-1 receptors, while downregulating ATP-binding cassette transporter A1 in macrophages (64). ATP citrate lyase (Acly) is a key metabolic enzyme that converts mitochondria-derived citrate into acetyl-CoA and oxaloacetate within the cytosol. Acly-dependent acetyl-CoA incorporation into histone promotes chromosome accessibility and regulates both LPS and IL-4-induced macrophage activation. Jeroen et al. demonstrated that macrophage ATP citrate lyase deficiency stabilizes atherosclerotic plaques (65). Thus, TMAO can downregulate ATP-binding cassette transporter A1 in macrophages to exacerbate atherosclerosis. In response to environmental stimuli, macrophages can be activated into several subtypes involved in different functional features, including two opposing phenotypic states with pro-inflammatory M1 and anti-inflammatory M2 cells. Similarly, TMAO induces M1 polarization of bone marrow-derived macrophages by nucleotide binding to oligomerization domain-like receptor protein 3 (NLRP3), which aggravates acute graft vs. host disease (GVHD) (66). Thus, TMAO aggravates the formation of atherosclerotic plaques by aggravating the inflammatory immune response of macrophages and their transformation to foam cells.

### Atherosclerosis: TMAO Activates ERS

The ER, formed in continuity with the outer membrane of the nuclear envelope, plays a key role as a central eukaryotic membranous organelle and is responsible for the synthesis, folding, and maturation of membrane and secreted proteins, lipid biosynthesis, and calcium storage (67). Endoplasmic reticulum stress is a state in which the accumulation of unfolded/misfolded proteins is accelerated after ER disorders caused by a variety of physiological and pathological conditions. Unfolded protein response (UPR) is an adaptive response of the ER to maintain homeostasis (68). There are three different UPR signaling pathways in ERS response that are mediated by (1) RNA-dependent protein kinase-like ER kinase (PERK), (2) activated transcription factor 6 (ATF6), and (3) inositol-required enzyme 1 (IRE1) (69). Increasing research on ERS has proven that it plays an important role in the development of various cardiovascular diseases (70). TMAO protects the structural and functional proteins of cells from damage by denaturing agents such as high osmotic pressure, hydrostatic pressure, NaCl, urea, or high temperature (19). Makhija et al. confirmed that TMAO, as a chemical molecular chaperone, has a protective effect on patients with early-stage asthma by reducing the expression of C/EBP homologous protein (CHOP) and resolving the maladaptive UPR signature (71). TMAO may induce defects in synaptic plasticity through the PERK signaling pathway mediated by ERS. A recent study showed that TMAO induces osteogenic responses in human aortic valve interstitial cells *via* ER–mitochondrial stress *in vitro* and aggravates aortic valve lesions in mice (72). In conclusion, ERS aggravates the progression of atherosclerosis, while TMAO can selectively bind to the PERK receptor. It is speculated that TMAO may aggravate the formation of atherosclerotic plaque by activating the PERK pathway and activating ERS.

### Thrombus Formation: TMAO Promotes Platelet Reactivity

Normal platelet function is essential for hemostasis and maintenance of a closed circulatory system. Platelet activation, aggregation, and subsequent intra-arterial thrombosis are important steps in atherosclerotic thrombosis, and enhanced platelet reactivity is associated with the degree of terminal organ injury and poor prognosis (73). Therefore, if the reactivity of the thrombus increases in atherosclerosis, the patient is more likely to have an embolism. Wang et al. (7) and others (74, 75) have found that TMAO can increase platelet reactivity. Zhu et al. showed that elevated TMAO levels can predict the risk of thrombotic events in humans. Platelet exposure to TMAO enhanced stimulus-dependent platelet activation through increased Ca^2+^ release from intracellular stores (76). Dietary choline, TMAO, and intestinal microorganisms *in vivo* can potentially lead to thrombosis, and within the physiological range of TMAO concentration observed in human and animal models, the effect of TMAO on platelet function is dose-dependent. However, the latest study provides a novel idea for the mechanism of TMAO promoting thrombosis, i.e., an increase in TMAO results in promotion of tissue factor (TF) expression in the vascular endothelium; thus the TF-dependent pro-thrombotic effect is pronounced (77). Interestingly, animal model studies employing dietary choline or TMAO, germ-free mice and microbial transplantation confirmed a role for gut microbiota-dependent TMAO production in modulating platelet hyperresponsiveness and thrombotic potential (78). This research provides new ideas for the subsequent prevention of thrombosis. At present, the main drugs used to prevent thrombosis in clinical practice are anti-steroids such as aspirin. Studies have shown that low-dose aspirin can inhibit the increase of TMAO in the body, along with reducing the platelet overreaction caused by increased TMAO (79). It is worth mentioning that the concept of thrombo-inflammation in atherosclerosis was proposed recently, which means that cell adhesion molecules not only accelerate the atherosclerotic process but also promote thrombosis (80). Moreover, activated platelets can trigger leukocyte adhesion and accumulation. Common antithrombotic treatments seem to reduce the inflammatory process. However, whether TMAO plays a role in this has not been reported thus far and merits further investigation.

### Cholesterol Metabolism: TMAO Reverses Cholesterol Transport and Inhibits Bile Acid Synthesis

**Figure 3:**
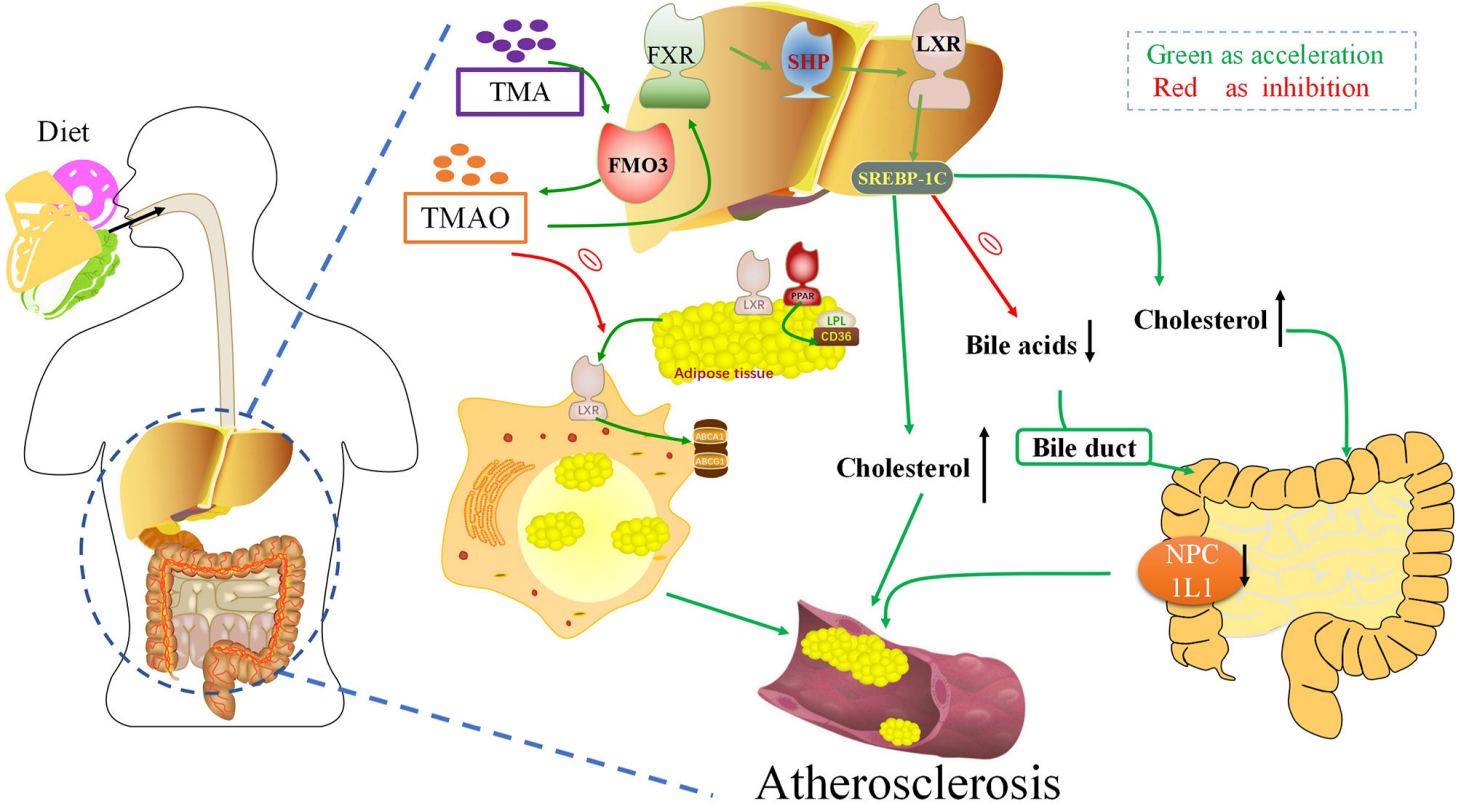
Trimethylamine-N-oxide (TMAO) can aggravate atherogenesis by altering bile acid and cholesterol metabolism. TMAO inhibits the RCT response, which inhibits the deposition of excess cholesterol in macrophages. TMAO can activate the nuclear receptors FXR and SHP, to reduce the expression of Cyp7a1 to inhibit bile acid synthesis, thus accelerating the formation of atherosclerosis. FXR, Farnesoid X receptor; SHP, Small heterodimeric partner; LXR, Liver X receptors; SREBPs, Sterol regulatory element-binding proteins; FMO3, Flavin-containing monooxygenase 3; NPC1L1, Niemann–Pick C1-like 1; TMA, Trimethylamine.

Deposition of cholesterol-rich lipoproteins is a common cause of atherosclerosis on the walls of blood vessels. Reverse cholesterol transport (RCT) is a process by which macrophages counteract the accumulation of excess cholesterol (81) (Figure 3). RCT mediates the outflow of cholesterol from foam cells accumulated in the atherosclerotic intima and has an anti-atherosclerotic effect in the early stage of atherosclerosis. In 2011, Wang et al. found that TMAO can inhibit the RCT process and affect cholesterol metabolism (7). TMAO can change the bile acid spectrum to accelerate the formation of aortic lesions in *ApoE*^−/−^ mice, and further activate the nuclear receptors farnesoid-X-receptor (FXR) and small heterodimer partner (SHP), to reduce the expression of cholesterol 7α-hydroxylase (Cyp7a1) to inhibit bile acid synthesis, thus accelerating the formation of atherosclerosis (82). Pathak treated an atherosclerotic mouse model with iodomethylcholine (IMC), a mechanism-based inhibitor of choline TMA lyase, and the loss of neutral sterols in mice feces was identified in the form of *coprostanol* (a bacterial metabolite of cholesterol) (83). In parallel, IMC treatment resulted in marked reductions in the intestinal sterol transporter Niemann–Pick C1-like 1 (NPC1L1) and reorganization of the gut microbial community, primarily reversing choline-supplemented diet-induced changes. IMC also prevented diet-driven hepatic cholesterol accumulation and caused both upregulation of the host hepatic bile acid synthetic enzyme CYP7A1 and altered the expression of hepatic genes critical for bile acid feedback regulation. TMA can be catalyzed as TMAO by flavin mono-oxygenase 3 (FMO3), a key rate-limiting enzyme in the liver. Studies have shown that reducing the content of FMO3 in insulin-resistant mice can inhibit FoxO1 (the central node of metabolic control), reduce the production of TMAO, and completely prevent the development of hyperglycemia, hyperlipidemia, and atherosclerosis (84). These studies suggest that the TMAO pathway driven by intestinal flora is closely related to microbial and host sterol and bile acid metabolism. Therefore, TMAO can aggravate atherosclerosis formation by affecting the metabolism of bile acid and cholesterol.

## Potential Therapeutic Measures of TMAO for AS

### Reduced Production of TMAO to Protect the Heart

**Figure 4:**
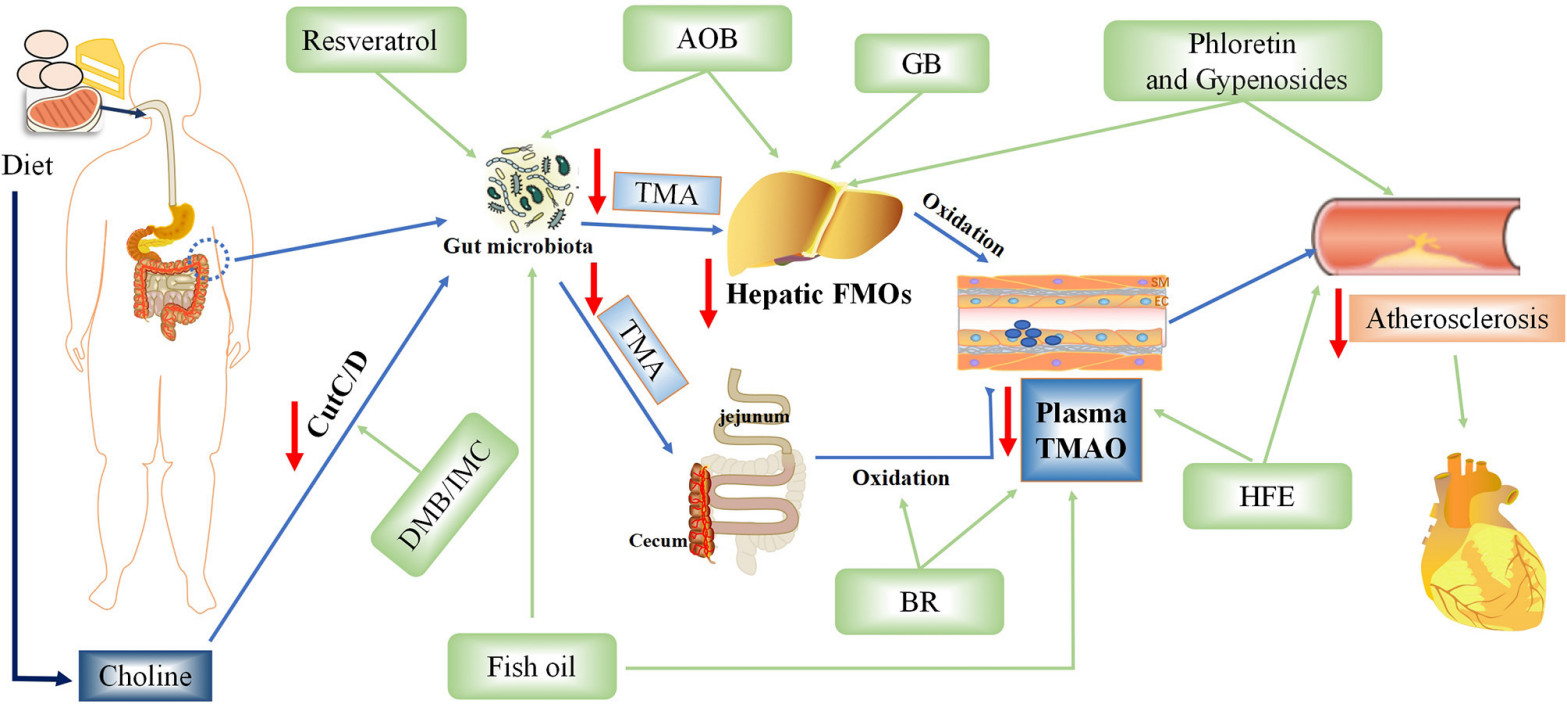
Potential therapeutic measures of Trimethylamine-N-oxide (TMAO) for atherosclerosis. AOB, *Alisma orientalis* beverage; GB, ginkgolide B; IMC, iodomethylcholine; BR, blackberry; HFE, hawthorn fruit extract; DMB, 3,3-dimethyl-1-butanol.

TMAO is an intestinal microbiota-dependent metabolite, which is accumulated in the body by ingestion of large amounts of substances rich in phosphatidylcholine. Recent studies have demonstrated that inhibiting various steps of TMAO production can reduce TMAO levels and treat AS (9, 85–88) (Figure 4).

#### Foods Rich in Phenols (Blackberry, Hawthorn Extract, and Resveratrol) Reduced TMA and Serum TMAO Levels

Polyphenols in blackberry (BR), especially anthocyanins, have many biological activities. Stable intake of BR extract can reduce hypercholesterolemia and hepatic inflammation induced by excessive choline in a high-cholesterol diet *via* decreasing the levels of cecal TMA and serum TMAO in rats (9). However, it is unclear which exact component of BR is responsible for this action. Hawthorn fruit is a bright red berry of the *Hawthorn* genus, in which phenolic compounds such as (-)-epicatechin, hyperin, and isoquercitrin are considered effective ingredients (85). Hawthorn fruit extract (HFE) can reduce atherosclerosis and inflammation aggravated by TMAO in a dose-dependent manner. HFE can also reverse the TMAO-induced decrease of antioxidant capacity by upregulating the expression of antioxidant enzymes including superoxide dismutase 1 (SOD1), SOD2, glutathione peroxidase 3 (GSH-Px3), and catalase (CAT) in the liver. Resveratrol, known as trans-3,4,5-trihydroxy-stilbene, is a type of biological polyphenol, mainly derived from peanuts, grapes, and mulberry. Resveratrol can regulate TMAO synthesis and bile acid metabolism by regulating the remodeling of intestinal flora, thereby reducing TMAO-induced atherosclerosis (86).

#### Ginkgolide B Can Inhibit the MRNA Level and Protein Expression of FMO3 and Reduce TMAO

Ginkgolide B is an herbal component of the extract obtained from *Ginkgo biloba* (GB) leaves (87). Studies have shown that ginkgolide can reduce dyslipidemia, inflammation, and intestinal barrier dysfunction in *ApoE*^−/−^ mice fed with a high-fat diet. At the same time, it can cause changes in the regulation of intestinal flora, especially an increase of *Bacteroides* species and decrease of *Helicobacter pylori*. Furthermore, *ApoE*^−/−^ mice treated with GB significantly inhibited the mRNA level and protein expression of FMO3, and then decreased the concentration of TMA and TMAO, thus slowing down the progression of atherosclerosis. However, it is not clear whether ginkgolide B protects the heart simply by reducing the production of TMAO.

#### Fish Oil Reduces TMAO Levels and Protects the Heart

It is worth mentioning that adding fish oil to the diet can improve TMAO-induced glucose-tolerance abnormalities, peripheral tissue insulin signal transduction, and adipose tissue inflammation to provide a novel therapeutic approach for metabolic diseases and reduce the population vulnerable to coronary atherosclerosis (89). However, a previous study found that fish can produce a large amount of TMAO, whose accumulation will increase the risk of cardiovascular disease (90). The protective effect of fish oil may be mediated by omega-3 fatty acids, which may outweigh any adverse effects of simultaneous production of TMAO *via* eating fish, showing a cardioprotective effect. Another study also confirmed that fish oil is more effective than flaxseed oil in regulating intestinal microflora and reducing TMAO-exacerbated atherogenesis (88).

### Protective Effect of Chinese Traditional Medicines on the Endothelium

#### Endothelial Protective Effect of Ketones

Choline, a precursor of TMAO, has been shown to reduce endothelial damage *via* its anti-inflammatory properties. Phloretin is a dihydrochalcone flavonoid commonly found in apples that has multiple biological activities, including vascular nutrition. Studies have confirmed that phloretin has a protective effect on hepatotoxicity and endothelial dysfunction induced by a high choline diet in mice (91). The main active components of the plant species *Gynostemma pentaphyllum* are *G. pentaphyllum* saponins. More than 100 kinds of *G. pentaphyllum* saponins have been isolated and identified. Studies have shown that saponins from this species have beneficial effects in the treatment of metabolic and vascular diseases. The same study confirmed the protective effect of the enrichment site of *G. pentaphyllum* saponins on vascular endothelial dysfunction and liver injury induced by high choline in mice (92). Tartary buckwheat flavonoids are an extract of Tartary buckwheat. Studies have shown that Tartary buckwheat flavonoids have protective effects on vascular dysfunction and liver injury induced by TMAO in mice, mainly through antioxidant stress, but the specific mechanism remains unclear (93).

#### *Alisma orientalis* Beverage, a Traditional Chinese Medicine, Reduces the Production of TMAO and Protects the Heart

*Alisma orientalis* beverage *(AOB)* is a traditional Chinese medicine consisting of a diverse medicinal plants; it has been used to treat metabolic syndrome and AS for a long time (94). The intestinal flora of mice treated with *AOB* was significantly different from that of mice fed high-fat diet. Additionally, *AOB* also decreased serum TMAO and hepatic FMO3 expression. This suggests that *AOB* may play a potential role in cardiovascular protection by reducing TMAO production and slow down the process of atherosclerosis. However, the main mechanism of this traditional Chinese medicine is still unclear; moreover, as individual heterogeneity is very obvious, more data is needed to support it.

### Intestinal Flora Modulators or Changes of Intestinal Flora Can Delay Atherosclerosis

Wang et al. showed that TMAO production was dependent on the gut microbiota and that mice treated with antibiotics failed to provide the required gut microbiota for TMAO production (7). It has been confirmed that changing the distribution or abundance of intestinal flora can reduce the production of TMAO and protect the heart. Later, Wang et al. discovered a non-lethal inhibitor drug −3,3-dimethyl-1-butanol (DMB)—which can promote the formation of endogenous macrophage foam cells and the development of atherosclerotic lesions in *ApoE*^−/−^ mice without changing circulating cholesterol levels (75). Pathak treated an atherosclerotic mouse model with the mechanism-based choline TMA lyase inhibitor IMC, a drug similar to DMB, which can prevent the diet-driven accumulation of cholesterol and change the expression of hepatic genes essential for bile acid feedback regulation (49). A novel oral carnitine challenge test (OCCT) was recently developed, in which the detection of *Ihubacter massiliensis* and/or *Emergencia timonensis* in the human gut can synergistically contribute to diagnosing high-TMAO producers, with 43% sensitivity and 97% specificity (95).

## Conclusion

In recent years, there has been an increase in knowledge about the intestinal flora, which acts as a large endocrine organ. In addition to the various resident bacteria performing different functions, the intestinal flora's active metabolites also have complex functions. Gut microbe-derived metabolites have been increasingly proposed as key factors to explore in cardiovascular health and disease research. Therefore, the intestinal flora can provide new ideas and approaches for the treatment and the healthy diet management of patients with chronic cardiovascular disease. TMAO is a classic chemical active molecule derived from the intestinal flora, and its role *in vivo* is relatively complex. Research has shown that it is non-toxic and even cardioprotective, possibly due to its ability to stabilize protein structure and the osmotic pressure in cells. However, its potential direct toxicity on the heart remains controversial and needs further investigation. In the latest international pooled analysis, the adverse associations of TMAO with certain cardiometabolic biomarkers have been elucidated, and the mechanisms of action, independent of renal function, warrant further investigation (96). The mechanism by which TMAO causes atherosclerosis will help us design appropriate drugs for optimum cardioprotection and better guide the diet of patients with myocardial infarction or coronary heart disease.

## Author Contributions

BYW and JQZ designed the manuscript. BYW wrote the manuscript. JQ revised the manuscript. JFL revised the tables. XY revised the figures. All authors approved the manuscript for publication.

## Conflict of Interest

The authors declare that the research was conducted in the absence of any commercial or financial relationships that could be construed as a potential conflict of interest.

## Publisher's Note

All claims expressed in this article are solely those of the authors and do not necessarily represent those of their affiliated organizations, or those of the publisher, the editors and the reviewers. Any product that may be evaluated in this article, or claim that may be made by its manufacturer, is not guaranteed or endorsed by the publisher.
